# When Stroke Speaks First: A Case Report on the Hidden Face of Streptococcus mitis Endocarditis

**DOI:** 10.7759/cureus.115676

**Published:** 2026-09-02

**Authors:** Christopher Fritzsche, Luca Bettini, Marco Bongiovanni

**Affiliations:** 1 Department of Internal Medicine, Ente Ospedaliero Cantonale, Lugano, CHE; 2 Department of Internal Medicine, Division of Infectious Diseases, Ente Ospedaliero Cantonale, Lugano, CHE

**Keywords:** cardiac surgery, cerebral infarction, heart tumor, infective endocarditis, streptococcus mitis, stroke

## Abstract

Multiple cerebral infarctions and intraparenchymal hemorrhages are typical complications of infective endocarditis, which can be easily misinterpreted as an indeterminate cardiac mass through non-invasive diagnostics.

We present a case of a 60-year-old man with acute dysarthria, jaw clonic movement, and gaze deviation, resulting from multiple cerebral infarctions caused by infective subacute endocarditis by *Streptococcus mitis,* confirmed by histopathological examination after surgical heart valve resection. By means of this case, we remind clinicians of the importance of investigating infective endocarditis in any patient with unexplained multiple ischemic strokes and an indeterminate heart tumor. Medical history taking, systematic clinical examination, and a multidisciplinary approach, including heart surgery, are essential for a good diagnosis, treatment, and prognosis.

## Introduction

Infective endocarditis (IE) is a potentially life-threatening infection of the endocardial surface of the heart, with an estimated incidence of approximately 3-10 cases per 100,000 person-years [1-3]. Neurological complications are among its most important extracardiac manifestations and may occur before or at the time of IE diagnosis, with cerebral embolization and ischemic stroke sometimes representing the first clinical presentation of the disease [1-3].

Early recognition of IE is essential because prompt antimicrobial treatment and appropriate surgical management can reduce the risk of further embolic events and other complications [2,4,5]. The decision to perform cardiac surgery in patients with neurological complications is complex and depends on several factors, including heart failure, uncontrolled infection, the risk of recurrent embolization, the characteristics of the cardiac lesion, and the presence or absence of intracranial hemorrhage [6-10]. However, diagnosis may be particularly challenging when IE presents predominantly with neurological manifestations and without fever or other typical systemic signs of infection.

Cardiac imaging may further complicate the diagnostic process when an infectious lesion has an atypical appearance. Although echocardiography remains the cornerstone of cardiac imaging in suspected IE, vegetations and inflammatory lesions may occasionally mimic intracardiac tumors or organized thrombi, while neoplastic lesions may similarly resemble infective vegetations [11-17]. In such situations, multimodality imaging may still fail to establish the nature of the lesion, particularly when blood cultures are initially negative.

We report a case of *Streptococcus mitis* infective endocarditis presenting predominantly with recurrent systemic embolization and multiple ischemic cerebral lesions, in which multimodality cardiac imaging suggested a neoplastic left ventricular mass and the initial blood cultures were repeatedly negative. The diagnosis was ultimately established through surgical exploration, histopathological examination, and molecular identification of* S. mitis*. This case highlights the diagnostic challenges of an atypical cardiac presentation of IE and the importance of maintaining a high clinical suspicion when multiple embolic events occur despite inconclusive non-invasive investigations.

## Case presentation

A 60-year-old Caucasian man was admitted to the emergency department because of acute dysarthria, jaw clonic movements, and deviation of the gaze to the right. On admission, blood pressure was 154 mmHg, body temperature was persistently normal, and neurological examination showed binocular paralysis of horizontal leftward gaze and dysarthria, corresponding to a National Institutes of Health Stroke Scale score of 2.

A few weeks before the current admission, the patient had experienced peripheral ischemia due to distal embolization of the left ulnar artery. A computed tomography (CT) scan performed at that time had also shown ischemic lesions involving the spleen and kidneys. These findings raised the suspicion of a systemic embolic process. The patient didn’t report any other chronic neurological, cardiac, or vascular diseases, recent surgical interventions, endoscopic procedures, or immunosuppressive conditions.

Initial laboratory investigations showed a marked elevation of lactate dehydrogenase, mild inflammatory syndrome, mild normocytic anemia, and mild renal impairment (Table 1).

**Table 1:** Pathological laboratory values eGFR: estimated glomerular filtration rate

Parameter	Result	Reference range
Hemoglobin	121 g/L	140-180 g/L
C-reactive protein	46 mg/L	< 5 mg/L
Creatinine	138 micromol/L	62-106 micromol/L
eGFR	48 mL/min/1,73m2	>60 mL/min/1.73m2
Lactic acid dehydrogenase	418 U/L	135-225 U/L

**Figure 1:**
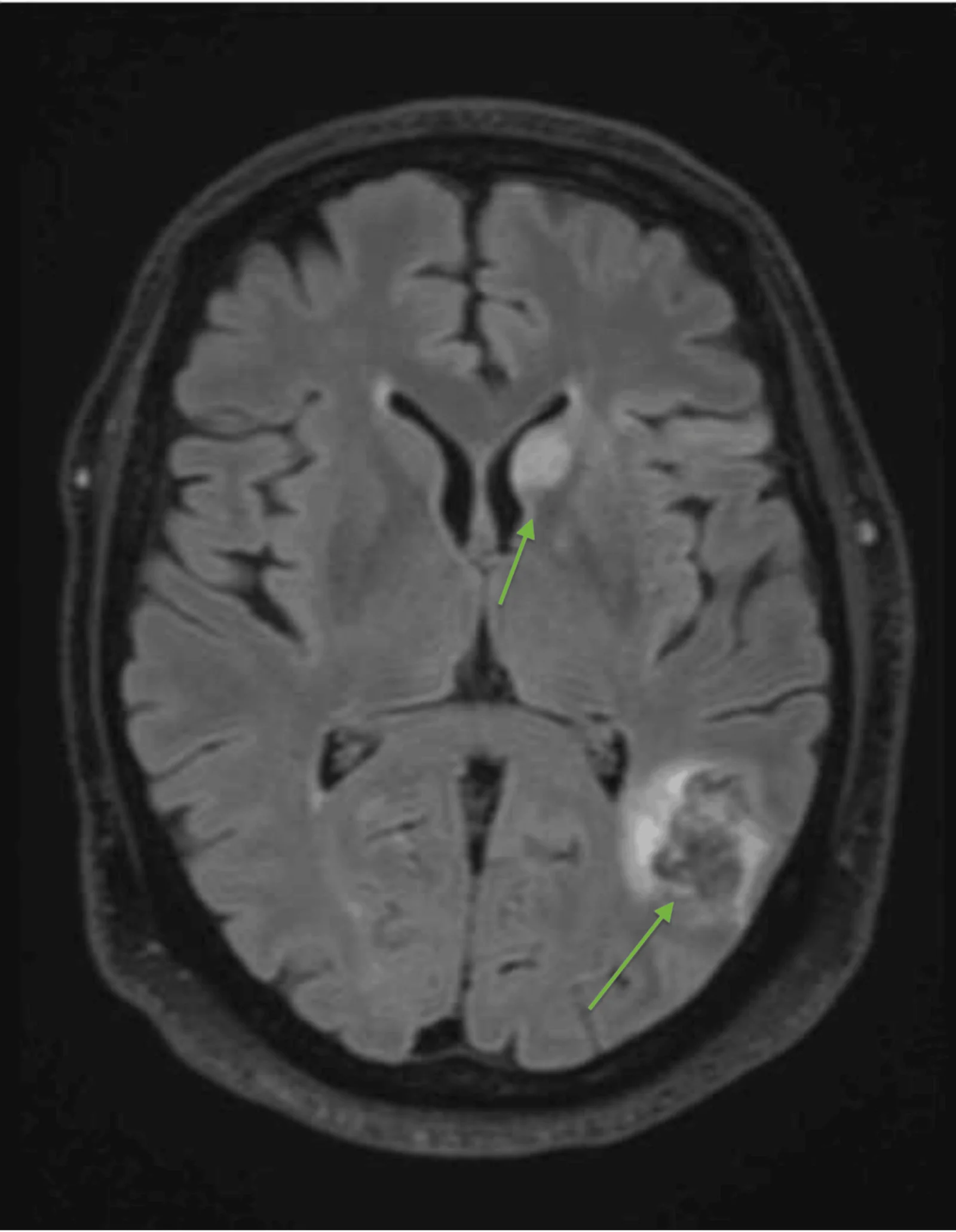
T2-weighted brain MRI showing multiple cortical ischemic lesions on the right and left cerebral hemispheres MRI: magnetic resonance imaging

Coagulation and autoimmune screening were unremarkable. Importantly, no antimicrobial therapy had been administered before the initial blood cultures were obtained. The first two sets of blood cultures were negative. Given the combination of systemic embolization and the absence of a clear source, an investigation for blood culture-negative endocarditis was performed, including serological testing for Brucella spp., *Coxiella burnetii*, *Bartonella henselae*, HIV, and syphilis, all of which were negative. Brain CT and magnetic resonance imaging (MRI) demonstrated multiple ischemic lesions involving different supra- and sub-tentorial vascular territories and at different stages of evolution, ranging from acute to late subacute lesions (Figure 1).

These findings confirmed the presence of multiple cerebral infarctions and, together with the previous ulnar, splenic, and renal ischemic lesions, supported the hypothesis of recurrent systemic embolization. Lumbar puncture showed no pleocytosis, while electroencephalography did not demonstrate epileptic activity. The patient was therefore admitted to the intensive care unit for neurological monitoring and further diagnostic evaluation.

**Figure 2:**
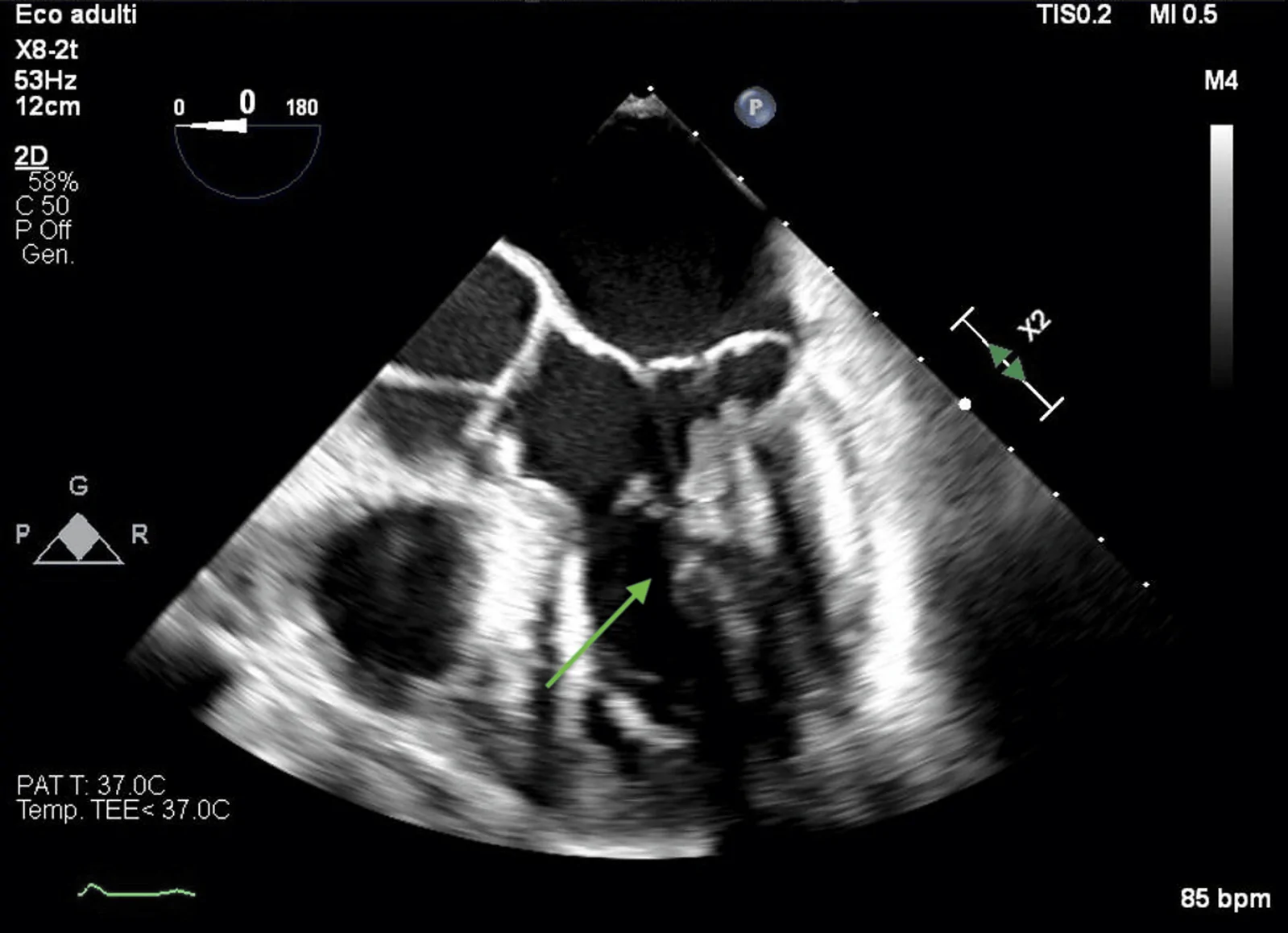
TEE showing mitral endocarditis as initially undetermined mass in the left ventricle TEE: transesophageal echocardiogram

Transthoracic echocardiography revealed a hyperechoic infiltrative lesion involving the basal anterior and anterolateral walls of the left ventricle. Transesophageal echocardiography subsequently demonstrated an approximately 2-cm mass with highly mobile and irregular margins, attached to the basal segments of the anterior, anterolateral, and lateral left ventricular walls, with apparent involvement of the adjacent endocardium and mitral sub-valvular region (Figure 2).

Because of its atypical appearance and uncertain anatomical origin, the lesion was considered potentially neoplastic, although an organized thrombus and an atypical infective lesion remained in the differential diagnosis.

**Figure 3:**
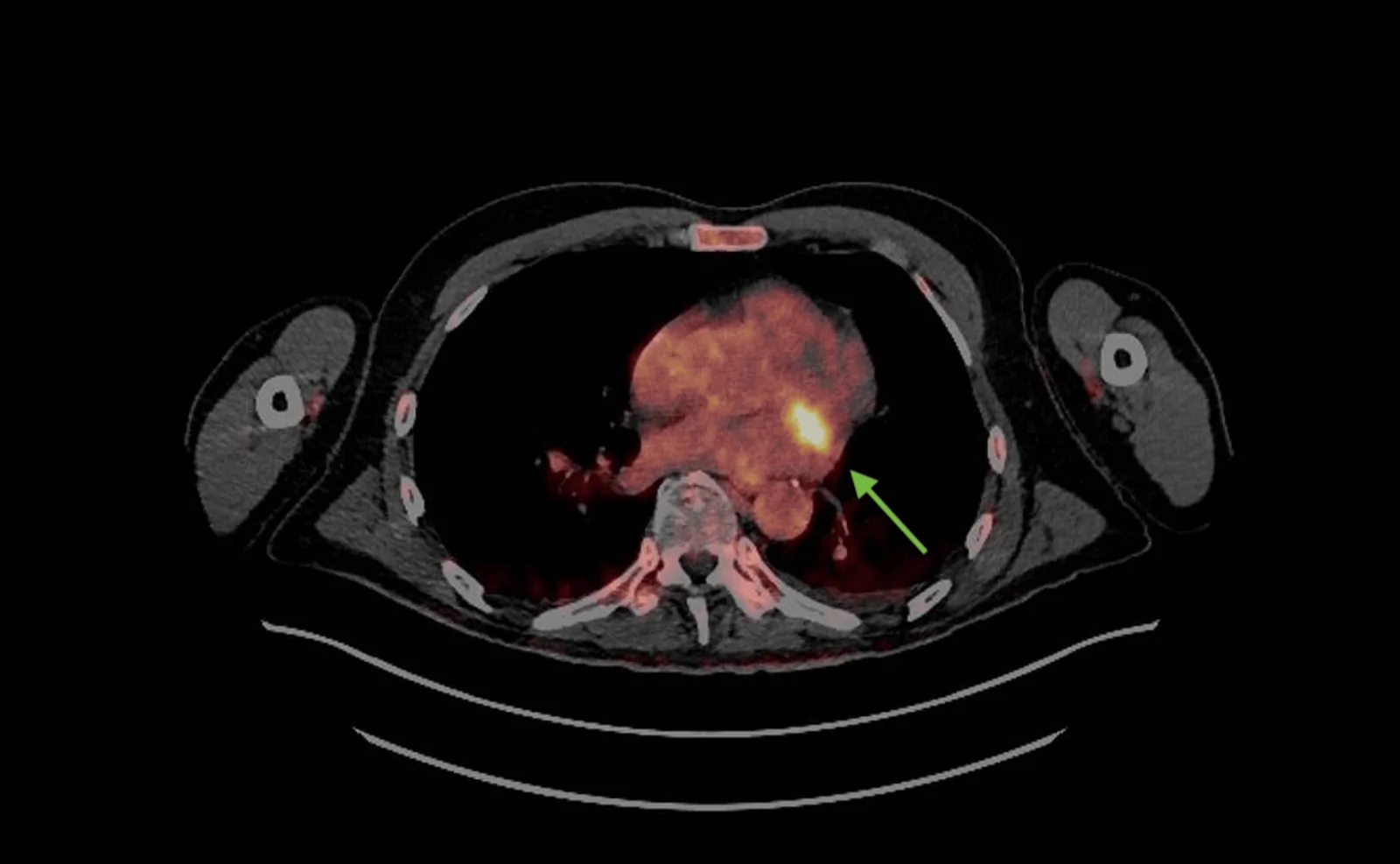
18F-FDG-PET/CT showing a lesion in the left ventricle with high metabolic rate 18F-FDG-PET/CT: fluorodeoxyglucose positron emission tomography/computed tomography

A multidisciplinary team involving neurologists, radiologists, infectious disease specialists, cardiologists, and cardiovascular surgeons reviewed the case. Given the unusual appearance of the cardiac lesion and the uncertainty regarding its nature, cardiac magnetic resonance imaging and whole-body 18F-fluorodeoxyglucose positron emission tomography/computed tomography (18F-FDG PET/CT) were performed. Cardiac MRI showed a probable neoplastic lesion associated with thrombus and lamellar calcification, located immediately below the mitral valve plane within the left ventricle. The 18F-FDG PET/CT demonstrated increased metabolic activity of the lesion but did not identify additional extracardiac sites of infection or malignancy (Figure 3). Despite this extensive multimodality imaging assessment, the nature of the cardiac lesion remained uncertain.

During his hospitalization, the patient experienced further episodes of neurological deterioration, considered clinically suggestive of recurrent cerebral embolization. In the absence of intracranial haemorrhage and given the recurrent systemic embolic events, the highly mobile cardiac lesion, and the persistent diagnostic uncertainty, the multidisciplinary team decided to proceed with surgical exploration. The patient underwent excision of the cardiac mass and mitral valve replacement with a bioprosthetic valve. The surgeon identified a subvalvular mitral mass at the P1 level, involving the mitral annulus and the anterolateral papillary muscle. The mass contained a cavity measuring approximately 1 × 1.5 cm, filled with gelatinous liponecrotic material, and partially communicating with the left ventricular cavity. The mitral valve appeared partially myxomatous.

**Figure 4:**
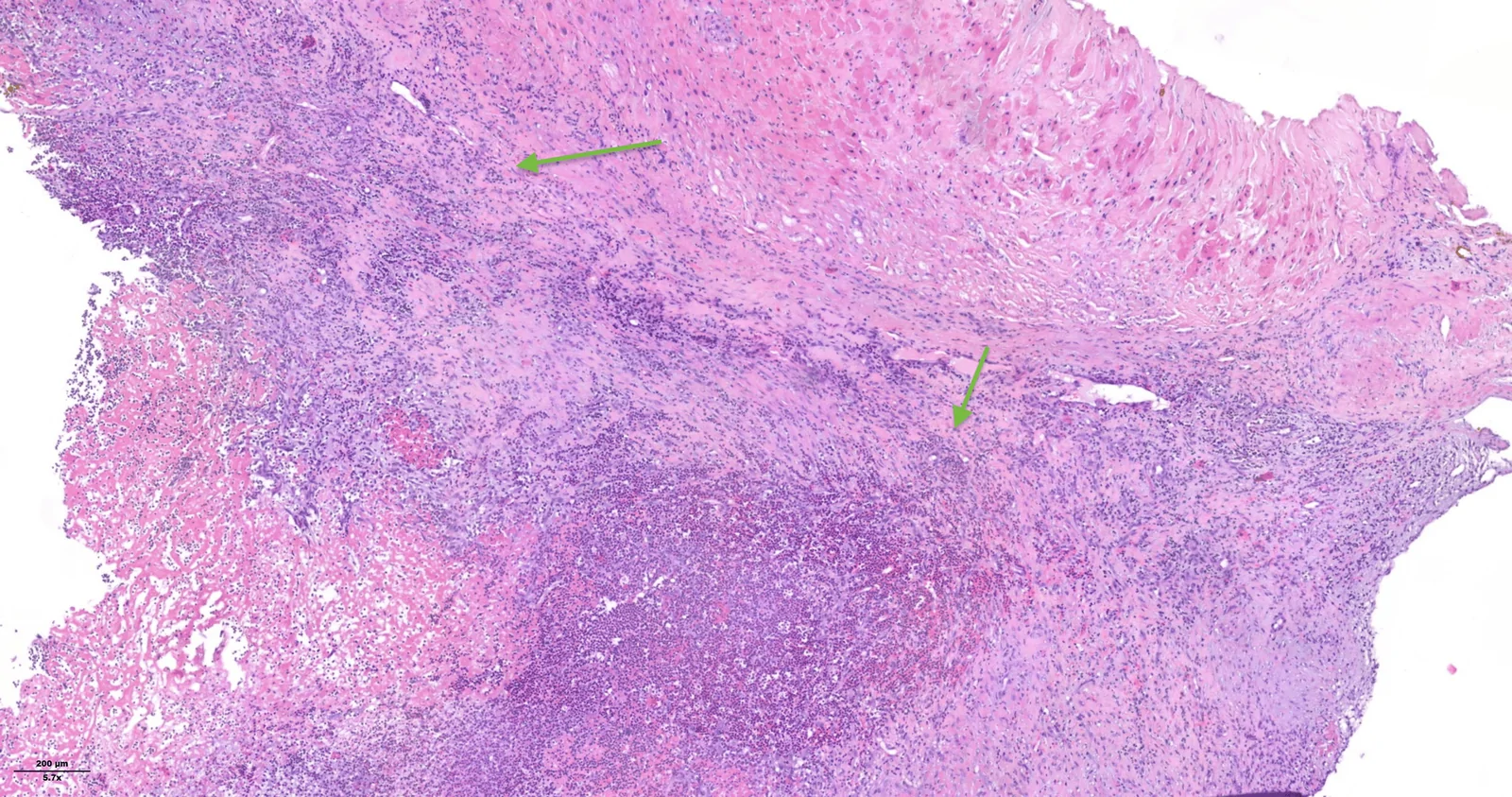
Section of valvular/endocardial tissue showing fibrinopurulent inflammatory exudate, necro-inflammatory debris, and underlying granulation tissue with fibroblastic proliferation and neovascularization (H&E, x5) H&E, x5: Hematoxylin and Eosin staining, 5x objective lens magnification

Histopathological examination of the surgical specimen demonstrated ulcerative and purulent endocarditis with extension of the inflammatory process into the adjacent myocardium and endocardium (Figure 4).

Whole-genome sequencing performed on the cardiac tissue identified *S. mitis*. In parallel, blood cultures were repeated every 48 hours after admission. On day 5, one of four blood culture bottles became positive for *S. mitis*. Because only one bottle was positive, this finding did not fulfil the major microbiological criterion of the 2023 Duke-ISCVID criteria [18], but it was consistent with the molecular identification obtained from the surgical specimen and with the histopathological evidence of active endocarditis.

**Figure 5:**
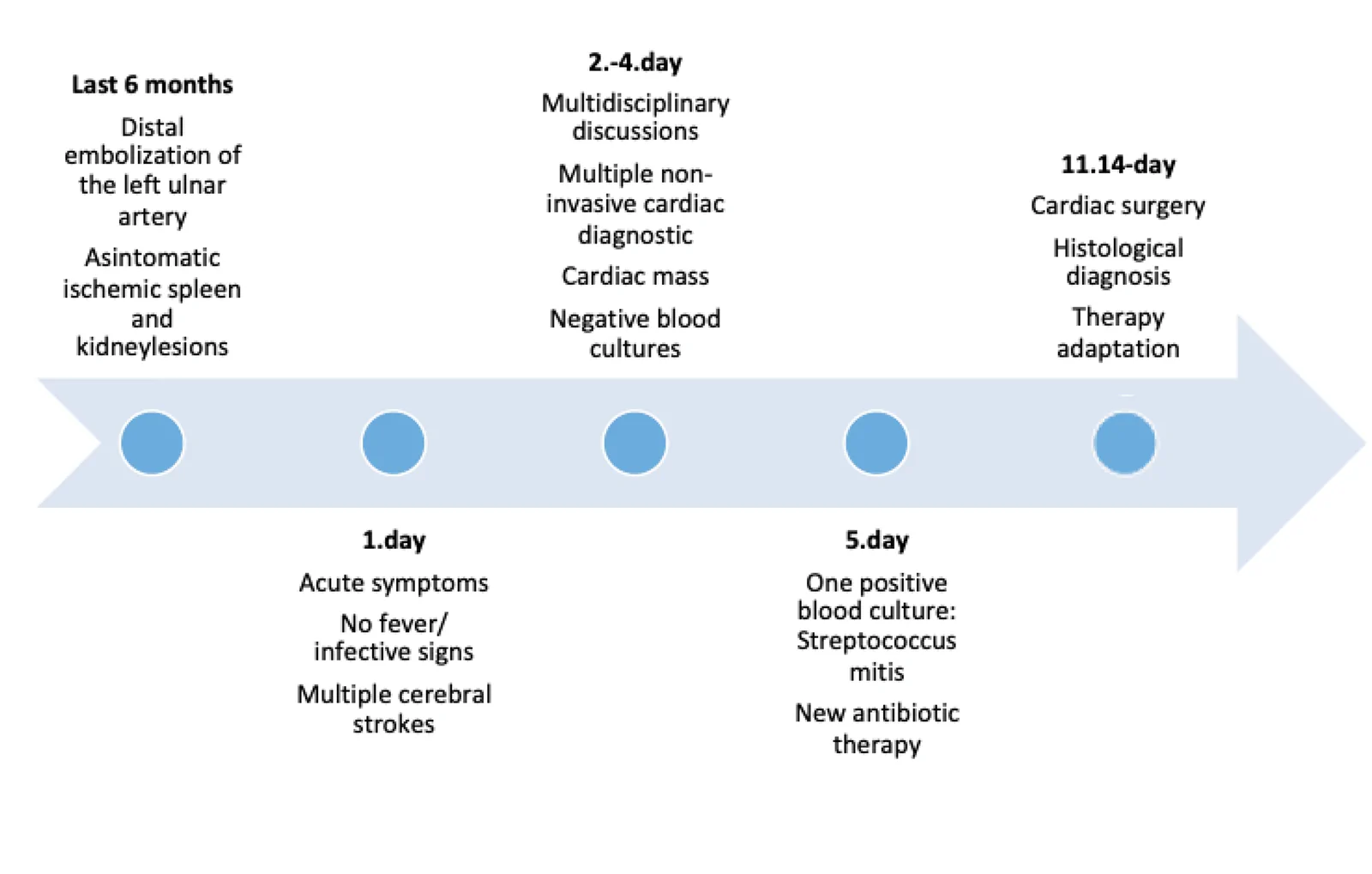
Timeline of events

Intravenous antimicrobial therapy was initiated with amoxicillin/clavulanic acid at a dose of 2.2 g every 6 hours and was continued for two weeks. Treatment was subsequently switched to ceftriaxone 2 g once daily for a further four weeks to facilitate early discharge and completion of therapy through an outpatient parenteral antimicrobial therapy programme. Surveillance blood cultures were repeatedly negative thereafter. The patient was discharged to neurological rehabilitation and remained free of recurrent neurological or cardiac complications during 12 months of follow-up. The timeline summarizes the chronological events (Figure 5). 

## Discussion

This case illustrates an unusual presentation of IE characterized by multiple systemic embolic events, an atypical tumor-like left ventricular cardiac lesion, absence of fever, and initially negative blood cultures. The diagnosis was ultimately established through surgical exploration, histopathological examination, and molecular identification of* S. mitis*. The case highlights the diagnostic challenges that may arise when IE presents predominantly with neurological manifestations and when cardiac imaging suggests a neoplastic rather than infectious process.

Neurological manifestations may be the first presentation of IE, particularly when cerebral embolization occurs before the development of classical systemic signs of infection [1,2,11]. In our patient, the initial presentation was neurological, but the previous ulnar artery embolization and concomitant splenic and renal ischemic lesions indicated a systemic embolic process. The presence of multiple ischemic lesions involving different vascular territories should therefore prompt consideration of a cardiac embolic source, including IE, even in an afebrile patient. In this context, the absence of fever should not be interpreted as evidence against IE when the overall clinical picture suggests systemic embolization.

The diagnostic process was further complicated by the unusual appearance of the cardiac lesion. Transthoracic and transesophageal echocardiography demonstrated an infiltrative and mobile mass involving the left ventricular endocardium and the mitral subvalvular region. Cardiac MRI initially favoured a neoplastic lesion with associated thrombus and calcification, while 18F-FDG PET/CT demonstrated increased metabolic activity but no additional extracardiac site suggestive of infection or malignancy. The main differential diagnoses therefore included infective vegetation/endocarditis, organized intracardiac thrombus, primary cardiac neoplasm, metastatic disease, and other inflammatory or calcified intracardiac lesions. Despite the use of multiple imaging modalities, the nature of the lesion could not be established with sufficient certainty.

Infective endocarditis may occasionally mimic an intracardiac tumor, while cardiac neoplasms and organized thrombi may similarly resemble infective vegetations [15-17]. This overlap is particularly relevant when the lesion has an unusual anatomical location or an infiltrative appearance. In our patient, the surgical and pathological findings demonstrated ulcerative and purulent endocarditis with extension of the inflammatory process into the adjacent myocardium and endocardium, correlating with the intraoperative finding of a subvalvular mitral mass at the P1 level, involving the mitral annulus and the anterolateral papillary muscle containing a cavity of approximately 1 x 1.5 cm filled with gelatinous liponecrotic material, communicating with the left ventricular cavity. If the lesion is confirmed to represent mural endocarditis, this would represent an uncommon manifestation, as IE more typically involves the valvular endocardium. The unusual anatomical presentation likely contributed to the initial suspicion of a cardiac neoplasm.

The microbiological diagnosis was also challenging. *S. mitis* is a recognized cause of bloodstream infection and IE and is typically associated with a subacute clinical presentation [12]. In our patient, however, the initial blood cultures were repeatedly negative despite the absence of previous antibiotic exposure. Only on day 5, one of four blood culture bottles became positive for *S. mitis*. This delayed and limited microbiological evidence contributed substantially to the diagnostic uncertainty. Possible explanations for initially negative blood cultures include low-level or intermittent bacteraemia, limited bacterial burden, or inadequate blood culture volume, although the precise reason remains uncertain.

According to the 2023 Duke-ISCVID criteria, a single positive blood culture for a typical microorganism causing IE does not fulfil the major microbiological criterion, which requires either two or more separate positive blood culture sets or persistently positive cultures. Thus, before surgery, the microbiological evidence in our patient was insufficient to establish a major microbiological criterion. The eventual identification of *S. mitis* in the surgical specimen by whole-genome sequencing provided important additional evidence of the infectious nature of the lesion. According to the 2025 AHA (American Heart Association) scientific statement on blood culture-negative endocarditis, nucleic acid-based detection of organisms other than *Coxiella burnetii*, Bartonella spp., or *Tropheryma whipplei *from cardiac tissue constitutes a minor criterion under the 2023 Duke-ISCVID criteria [14]. Nevertheless, in the present case, molecular identification of *S. mitis* in surgically excised tissue, together with histopathological demonstration of purulent endocarditis and the clinical evidence of systemic embolization, provided compelling evidence of IE. This case emphasizes the importance of obtaining multiple blood culture sets before antimicrobial therapy and repeating cultures when clinical suspicion remains high despite initially negative results.

Neurological and embolic manifestations have previously been described in patients with *S. mitis* infective endocarditis [12,19-21]. These reports, as well as our case, illustrate that viridans-group streptococcal IE may present with neurological symptoms before the diagnosis of IE is established. Cerebral embolization may dominate the clinical presentation, while fever or other systemic manifestations may be absent or delayed. Our case differs from previously reported presentations because the cardiac source of embolization appeared as an infiltrative left ventricular mass rather than a conventional valvular vegetation. In addition, the lesion showed imaging features suggestive of a cardiac neoplasm, and the initial microbiological workup was repeatedly negative. The combination of an atypical cardiac mass, multiple systemic emboli, and limited initial microbiological evidence therefore resulted in substantial diagnostic uncertainty and ultimately necessitated surgical exploration.

The case also highlights the importance of a multidisciplinary endocarditis team approach, as emphasized in the 2023 ESC (European Society of Cardiology) guidelines [10]. The involvement of neurologists, radiologists, infectious disease specialists, cardiologists, and cardiovascular surgeons allowed the clinical, neurological, microbiological, and imaging findings to be considered together. In particular, the decision to proceed to surgery was based on the recurrent embolic phenomena, the highly mobile cardiac lesion, and the inability of non-invasive investigations to establish its nature.

The timing of surgery represents another important aspect of this case. Current ESC recommendations support early or urgent surgery in patients with left-sided IE and a high risk of recurrent embolization, particularly in the presence of large or mobile vegetations and recurrent embolic events [6,10]. In patients with ischemic stroke, the absence of intracranial haemorrhage generally does not preclude early cardiac surgery when a strong cardiac indication is present. In retrospect, the recurrent neurological events and the highly mobile cardiac lesion could have supported earlier surgical intervention. However, the clinical decision was particularly challenging because IE had not been definitively established at that stage: the initial blood cultures were negative, the cardiac lesion had an atypical tumour-like appearance, and the patient did not initially fulfil the criteria for definite IE. Thus, the indication for surgery was based on the combination of recurrent embolic events, the potential risk of further embolization, and the uncertain nature of the cardiac mass rather than on a definitive preoperative diagnosis of IE. We cannot determine whether earlier surgery would have prevented the recurrent neurological events. Nevertheless, this case illustrates that recurrent systemic embolization from a highly mobile intra-cardiac lesion should prompt urgent multidisciplinary reassessment and consideration of surgical intervention, even when the diagnosis of IE remains uncertain.

Surgical intervention ultimately provided both diagnostic and therapeutic benefit. Histopathological examination demonstrated ulcerative and purulent endocarditis with extension of the inflammatory process into the adjacent myocardium and endocardium, while molecular testing identified S. mitis. The surgical approach therefore resolved the diagnostic uncertainty created by the atypical imaging appearance and limited microbiological evidence. The decision to replace the mitral valve rather than perform isolated excision of the mass was based on intraoperative findings. This information is relevant because it clarifies the anatomical extent of the infectious process and the rationale for the surgical strategy.

Overall, this case demonstrates that IE should remain in the differential diagnosis of patients presenting with multiple systemic embolic events and an indeterminate cardiac mass, even in the absence of fever or initially positive blood cultures. The combination of repeated blood cultures, multimodality imaging, careful clinical assessment, and multidisciplinary discussion is essential when diagnostic uncertainty persists. In selected patients with recurrent embolization and a highly mobile intra-cardiac lesion, early surgical assessment should be considered in accordance with current guideline recommendations. In our patient, surgical intervention allowed definitive characterization of the cardiac lesion and, together with antimicrobial therapy, was followed by clinical recovery without recurrent neurological or cardiac complications during 12 months of follow-up.

## Conclusions

Infective endocarditis (IE) is a potentially life-threatening disease with a broad and sometimes misleading clinical and radiological presentation. Our case illustrates that IE should remain in the differential diagnosis of patients presenting with multiple systemic embolic events and an indeterminate cardiac mass, even in the absence of fever or initially positive blood cultures. Repeated blood cultures, multimodality imaging, careful clinical assessment, and multidisciplinary discussion are essential when diagnostic uncertainty persists. In selected patients with recurrent embolization and a highly mobile intra-cardiac lesion, early surgical assessment should be considered in accordance with current guideline recommendations, even when the diagnosis of IE is not yet definitive.

In this case, surgical intervention allowed definitive characterization of the cardiac lesion and, together with antimicrobial therapy, was followed by clinical recovery without recurrent neurological or cardiac complications during 12 months of follow-up. The case highlights the value of an endocarditis team approach and of histopathological and molecular examination when conventional diagnostic investigations remain inconclusive.
